# The journey of a generation: advances and promises in the study of primordial germ cell migration

**DOI:** 10.1242/dev.201102

**Published:** 2024-04-12

**Authors:** Lacy J. Barton, Lorena Roa-de la Cruz, Ruth Lehmann, Benjamin Lin

**Affiliations:** ^1^ Department of Neuroscience, Developmental and Regenerative Biology, The University of Texas at San Antonio, One UTSA Circle, San Antonio, TX 78249, USA; ^2^ Whitehead Institute and Department of Biology, MIT, 455 Main Street, Cambridge, MA 02142, USA; ^3^ Department of Biochemistry & Cell Biology, Stony Brook University, Stony Brook, NY, 11794, USA

**Keywords:** ECM, Gonad, Migration, Primordial germ cells, Signaling

## Abstract

The germline provides the genetic and non-genetic information that passes from one generation to the next. Given this important role in species propagation, egg and sperm precursors, called primordial germ cells (PGCs), are one of the first cell types specified during embryogenesis. In fact, PGCs form well before the bipotential somatic gonad is specified. This common feature of germline development necessitates that PGCs migrate through many tissues to reach the somatic gonad. During their journey, PGCs must respond to select environmental cues while ignoring others in a dynamically developing embryo. The complex multi-tissue, combinatorial nature of PGC migration is an excellent model for understanding how cells navigate complex environments *in vivo*. Here, we discuss recent findings on the migratory path, the somatic cells that shepherd PGCs, the guidance cues somatic cells provide, and the PGC response to these cues to reach the gonad and establish the germline pool for future generations. We end by discussing the fate of wayward PGCs that fail to reach the gonad in diverse species. Collectively, this field is poised to yield important insights into emerging reproductive technologies.

## Teaser

**Summary:** This Review covers the mechanisms of primordial germ cell migration in model organisms and systems, emphasizing the guidance factors and signaling pathways necessary for colonization of the gonad.

## Introduction

### The road well-traveled

Primordial germ cells (PGCs) are the precursors to egg and sperm, which give rise to the next generation. Essential to reproduction and the preservation of species, PGCs are often among the first cell types specified in the embryo, long before the development of the somatic cell types of the somatic gonad required for gamete production. Thus, across the animal kingdom, PGCs must migrate through many tissues to reach the developing testis and ovary. This migration often starts at the periphery of the blastula; this initial peripheral localization of PGCs reflects segregation of germ plasm in many species or peripheral localization of inductive PGC signals (Dixon, 2007). Indeed, even *in vitro*-derived PGC-like cells (PGCLCs) are found first at the periphery in embryoids (Mitsunaga et al., 2017). In animals, the peripheral specification of PGCs generally necessitates a long journey to the gonad.

**Fig. 1.**
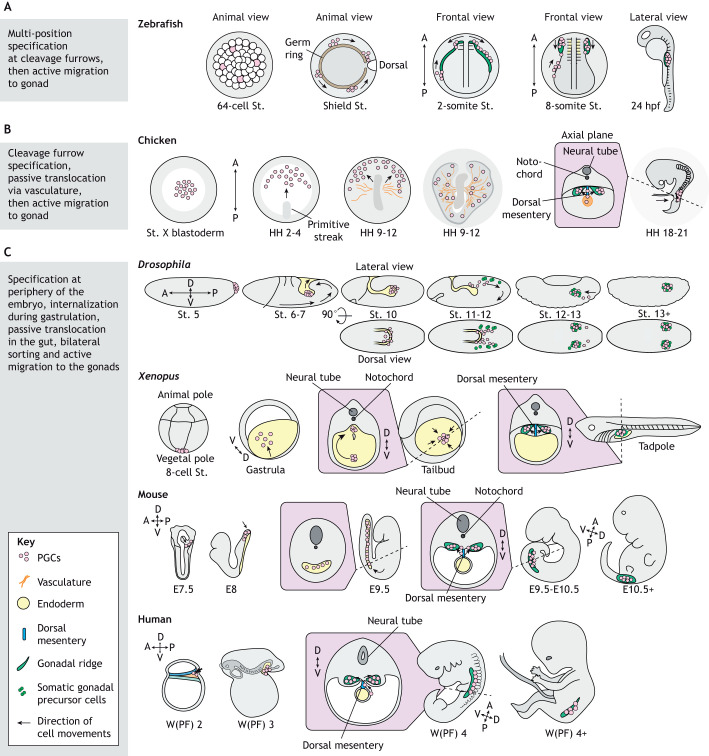
**Routes PGCs migrate along to reach the somatic gonad.** Three broad pathways are followed by PGCs among studied model organisms. (A) One path is exemplified by the zebrafish model *Danio rerio*, in which PGCs are specified at multiple locations in the early embryo, often at cleavage furrows. Then, PGCs actively migrate dorsally during the shield stage, converging from four populations into two bilateral groups. Then, at the somite stages, PGCs migrate laterally and anteriorly as they colonize the gonad. (B) A second PGC route is found in chicken (*Gallus gallus domesticus*). Like zebrafish, chicken PGCs are specified near cleavage furrows. In contrast to zebrafish, chicken PGCs reach the gonad through both passive translocation and active migration. First, PGCs move anteriorly toward the germinal crescent, where they enter blood vessels. After circulating, PGCs exit the vascular endothelia and actively migrate through the dorsal mesentery toward bilateral developing gonads. (C) A third, common PGC route is found in several model organisms, including *Drosophila melanogaster*, *Xenopus laevis* and mouse (*Mus musculus*). This is also the route that PGCs likely follow in humans, although the origin of human PGCs is as yet unclear. In *Drosophila*, *Xenopus* and mouse, PGCs are specified at the border of the embryo and extra-embryonic tissue. From this peripheral location, PGCs are passively internalized into the endoderm during gastrulation. Mouse PGCs actively move to the endoderm, but once associated with the hindgut epithelium they move passively with the gastrulating hindgut. From the gut, PGCs traverse the endodermal epithelium, entering the mesoderm (commonly the dorsal mesentery) where they actively migrate and sort bilaterally to reach the two gonadal ridges. In *Drosophila*, the germ-band, which contains the somatic gonadal precursors (green), retracts during PGC migration, depicted by the black arrows. Cell movements are indicated by black arrows. For all species, developmental progression is shown from left to right. Each embryonic stage is shown underneath the corresponding embryo schematic. A, anterior; D, dorsal; E, embryonic day; hpf, hours post-fertilization; HH, Hamburger and Hamilton stage; P, posterior; St., stage; V, ventral; W(PF), weeks post fertilization.

**Fig. 2.**
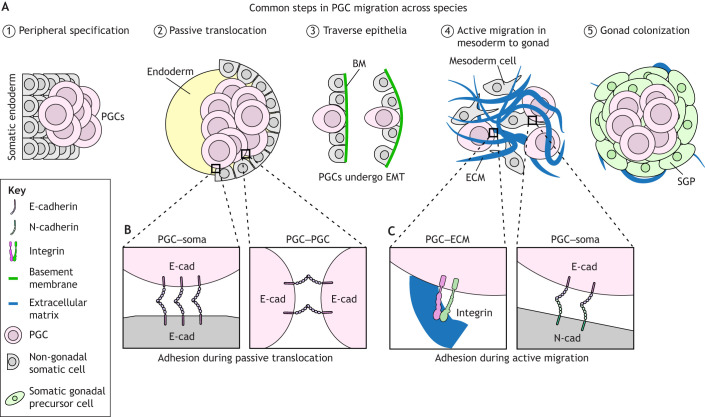
**Adhesion properties during common phases in PGC migration.** Schematic of the five stages of PGC migration in fruit flies and frogs. Mice follow the same stages, but actively migrate to enter the endoderm at Stage 2 and then continue to migrate within it. (A) Stage 1: PGCs are specified at the periphery of embryos or extra-embryonic regions early in embryogenesis. Stage 2: PGCs are passively translocated by gastrulation into the interior of the embryo, often with the future endoderm. Stage 3: PGCs transmigrate through an epithelial barrier to access the mesoderm. Stage 4: PGCs directionally migrate through mesoderm containing cells and extracellular matrix. Stage 5: PGCs are ensconced by the somatic component of the gonad. (B,C) Common cell–cell and cell–ECM adhesion molecules that PGCs use to reach the gonad. During passive translocation (B), PGC–PGC and PGC–somatic cell contacts are commonly mediated by E-cadherin (E-cad). During active migration (C), PGCs migrate upon other cells and ECM in the mesoderm. PGCs express E-cadherin early in development whereas mesoderm cells express N-cadherin (N-cad). This suggests PGCs may use heterotypic cadherin contacts (E-cadherin on PGC to N-cadherin on mesoderm) for cell-on-cell migration. PGCs also express integrin receptors to migrate on ECM. BM, basement membrane; ECM, extracellular matrix; EMT, epithelial-to-mesenchymal transition; SGP, somatic gonadal precursor.

Routes that PGCs use to reach the gonad vary among species; these are illustrated in Fig. 1 for common model organisms. In many animals, such as fruit flies, sea urchins and frogs, the journey begins with the passive translocation of PGCs during gastrulation (Figs 1 and 2A) (Nishiumi et al., 2005; Yajima and Wessel, 2012; DeGennaro et al., 2011). In the mouse, a combination of active and passive movements seems to facilitate internalization as newly specified mouse PGCs initially display features of active migration, but, once associated with the hindgut epithelium, they move in synchrony with the gastrulating hindgut (Anderson et al., 2000; Spiegelman and Bennett, 1973; Kanamori et al., 2019; Roelen and Chuva de Sousa Lopes, 2022). During or shortly after passive translocation, PGCs are often located within the endoderm, as observed in the fruit fly, sea urchin, mouse, rat and marmoset monkey (Kamimura et al., 1980; Molyneaux et al., 2001; Kemper and Peters, 1987; Aeckerle et al., 2015; Warrior, 1994; Jaglarz and Howard, 1994; Campanale et al., 2014) (Fig. 1C). Although common, an endodermal route is not universal. Zebrafish PGCs are not internalized into the gut, but rather associate with the endoderm toward the end of their journey (Braat et al., 1999; Paksa et al., 2016) (Fig. 1A). Instead, zebrafish PGC translocation is supported by the movements of underlying cells in the gastrulating embryo (Weidinger et al., 1999). Whereas chicken PGCs circulate through the vasculature system, recent work in quails suggests that avian PGCs passively enter the vasculature following envelopment by endothelial cells (Fig. 1B) (Meyer, 1964; Murai et al., 2021). Whether by vasculature, endoderm or other gastrulating cells, this early passive translocation provides an efficient mechanism to bring peripherally specified PGCs closer to the internally developing somatic gonads.

In the second stage of their journey, PGCs transition from passive translocation to active, directed migration toward the somatic gonad in many model organisms (Fig. 2A). This process often starts with PGC extravasation through an endo- or epithelial layer as a result of endodermal remodeling, as well as changes in cadherin expression, extracellular matrix (ECM) composition, and tissue stiffnesses along the migratory path (Fig. 2B) (Seifert and Lehmann, 2012; Baronsky et al., 2016; Dzementsei et al., 2013; De Felici et al., 2005; Saito et al., 2022; Paksa et al., 2016). Upon entering the mesoderm, PGCs in animals as diverse as fruit flies and mice sort into two populations by migrating laterally, and then each population migrates in a directed fashion toward one of two developing somatic gonads (Clark and Eddy, 1975; Fujimoto et al., 1977; Meyer, 1964; Moore et al., 1998; Heasman and Wylie, 1978). In animals such as chicken, zebrafish and mice, this migration occurs through the dorsal mesentery (Fig. 1) (reviewed by Hen and Sela-Donenfeld, 2019; Cantu and Laird, 2017). Although the somatic cell types that PGCs encounter during the final stage of their journey may differ across species (Mollgard et al., 2010; Sasaki et al., 2016; Wolff et al., 2019; Hoyer et al., 2005; Stepanik et al., 2016), final homing and PGC colonization is facilitated by attractive and repulsive chemical cues that collectively shepherd PGCs to their destination (summarized by Richardson and Lehmann, 2010). In this Review, we first discuss the adhesion, biophysical and chemical-based cues that surrounding somatic cells provide to migrating PGCs. We then review what is known about how PGCs in various species respond to environmental cues and what happens when PGCs fail to colonize the gonad. Finally, we discuss how studies of PGC migration in emerging model organisms and *in vitro* PGCLC models can advance both fundamental and translational reproductive biology.

## The somatic shepherd: Adhesion, stiffness, and chemical signals

### Cadherin-based cell–cell adhesions

PGCs are specified early in development, before the formation of elaborate embryonic structures and the extensive deposition of interstitial ECM. Thus, young PGCs chiefly adhere to and migrate on other cells (Fig. 2B). Central to these early cell interactions are cadherins, universal transmembrane Ca^2+^-dependent adhesive molecules, well known as components of adherens junctions in epithelial cells. Cadherins expressed in PGCs include epithelial (E)-cadherin and neural (N)-cadherin (also known as cadherin 1 and 2, respectively) (Table 1; see also Box 1 for information on how the data in Table 1 was collated). Homophilic cadherin bonds are essential for tissue cohesion and segregation during morphogenesis (Halbleib and Nelson, 2006) and fulfill similar roles early in PGC development (Okamura et al., 2003; DeGennaro et al., 2011; Blaser et al., 2005).

**Box 1. Approach to the compilation of data in Table 1**

In Table 1, we analyze the gene expression patterns and percentage of cells expressing each gene based on dataset type. For *Drosophila*, we used an online searchable single-cell RNA-sequencing (scRNA-seq) dataset, focusing on PGCs. For cynomolgus monkey and mouse, we analyzed the available scRNA-seq matrix using Seurat (Hao et al., 2021). For chicken, we generated single-cell feature counts from fastq files and calculated differentially expressed genes (DEGs). For *Xenopus*, hPGCLCs and human PGCs, we identified reads per kilobase million (RPKM)/transcripts per kilobase million (TPKM) values from bulk RNA-seq searchable tables. For zebrafish, we used bulk RNA-seq fastq files and calculated DEGs with DESeq2 (Love et al., 2014). Taken together, the data in Table 1 reveal trends of adhesion molecule expression in PGCs from different animals, showing that cadherin and integrin diversity and expression increases over time. There is no evidence of a cadherin switch, as seen in other developmental migration contexts (Scarpa et al., 2015; Nakagawa and Takeichi, 1995). It is important to note that post-transcriptional regulation is a hallmark of PGC biology, so these results will need to be validated at the protein level.

The source data have been previously published (Calderon et al., 2022; Rengaraj et al., 2022; Owens et al., 2017; Butler et al., 2018; D'Orazio et al., 2021; Zhao et al., 2021; Zhai et al., 2022; Irie et al., 2015) and can be accessed in Gene Expression Ominibus or ArrayExpress (for E-MTAB-8707) using the following accession numbers: GSE190149, GSE86146, GSE80971, GSE60138, E-MTAB-8707, GSE148032, GSE193007, GSE60138, respectively.

**Table 1.**
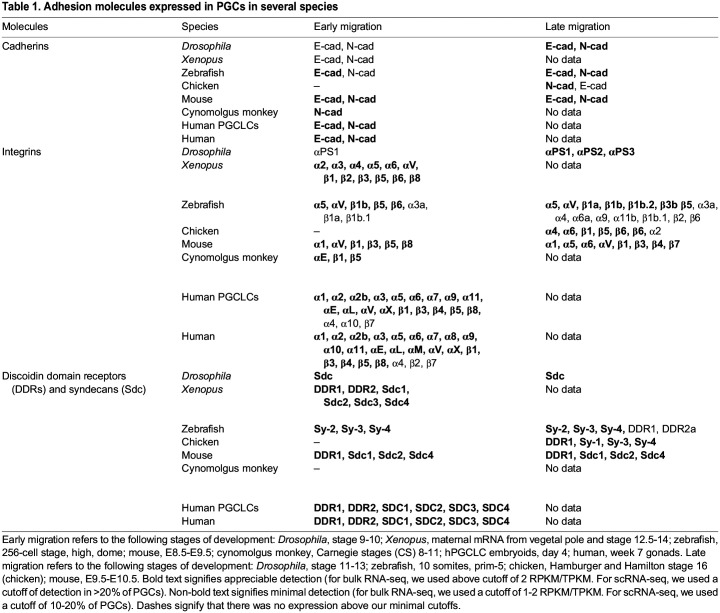
Adhesion molecules expressed in PGCs in several species

In many species, newly specified PGCs cluster with each other (Terayama et al., 2013; DeGennaro et al., 2011; Okamura et al., 2003; Muniesa and Dominguez, 1990; Weidinger et al., 1999; Yajima and Wessel, 2012), often in an E-cadherin-dependent manner (DeGennaro et al., 2011; Okamura et al., 2003; Chihara and Nance, 2012), preventing them from straying and receiving erroneous inductive signals (Okamura et al., 2003). Mouse PGCs, in contrast, are interspersed with other cells (Ohinata et al., 2005). The surrounding somatic cells also express E-cadherin in these early stages in flies (Oda et al., 1994), worms (Chihara and Nance, 2012), fish (Blaser et al., 2005) and frogs (Baronsky et al., 2016). Thus, young PGCs can hold on to each other and their somatic neighbors (Fig. 2B). In flies, these cadherin-mediated PGC–PGC and PGC–somatic cell bonds function in tandem to allow PGCs to ‘piggyback’ into the embryo interior during the morphogenetic movements of gastrulation (DeGennaro et al., 2011) (Fig. 1C), whereas only PGC–PGC adhesions are necessary for this to occur in worm PGCs (Chihara and Nance, 2012). We speculate that cadherins are universally required for passive PGC transit and may play a more active role in retaining PGCs in certain locations. Mouse PGCs travel through primitive streak mesoderm to reach the definitive endoderm (Gu et al., 2009). The retention of mouse PGCs in the endoderm may involve cadherin-dependent cell sorting, as the primitive streak mesoderm expresses N-cadherin, whereas the endoderm expresses both N and E-cadherin (Scheibner et al., 2021).

Once PGCs access the interior of the embryo, motile PGCs are developmentally restrained by an epithelial somatic barrier in flies (Seifert and Lehmann, 2012) and mice (Molyneaux et al., 2001), suggesting that somatic cells control the timing of PGC entry into new embryonic locales. Somatic cells may also control the entry of avian PGCs into the vascular system, as quail PGCs are ensconced by endothelial cell precursors prior to vascularization (Murai et al., 2021). This gatekeeping function is E-cadherin dependent in flies and relies upon a developmental epithelial-to-mesenchymal transition in the surrounding endoderm (Jaglarz and Howard, 1995), where adherens junctions break down and intercellular gaps form for migrating PGCs (Fig. 2A). Premature junction dissolution leads to precocious PGC invasion in flies (Seifert and Lehmann, 2012). In mice, the barrier may be the endoderm basal lamina, as PGCs seem to displace hindgut cells, linked by adherens junctions, from the basal lamina (Clark and Eddy, 1975; Molyneaux et al., 2001). Human PGCs also transiently reside within the hindgut and can be observed breaking through the hindgut basal lamina enroute to the gonadal ridges (Fujimoto et al., 1977). It remains unclear how mouse and human PGCs traverse the basal lamina, but based on other organisms or migration systems, possible mechanisms include the autonomous secretion of matrix metalloproteases, egression through existing gaps, physical displacement, or a combination of these processes.

Actively migrating PGCs encounter tissues expressing a varying repertoire of cadherins (Fig. 2C). After crossing through the E-cadherin-expressing endoderm, mouse, fly and frog PGCs meet the N-cadherin-expressing mesoderm and are in intimate contact with these cells (Zamboni and Merchant, 1973; Heasman et al., 1985; Richardson and Lehmann, 2010). Although mouse PGCs possess both E- and N-cadherin mRNAs at both stages (Table 1), antibody staining suggests that mouse PGCs express E-cadherin protein but do not express N-cadherin until they have ceased migrating (Bendel-Stenzel et al., 2000). Thus, these interactions may arise from heterotypic binding between E-cadherin on PGCs and N-cadherin on the mesoderm, analogous to their early contact with the primitive streak mesoderm and as observed between stromal and endoderm-derived cells (Straub et al., 2011), or through unidentified adhesion molecules. Although, to our knowledge, the effect of mesoderm-specific cadherin knockdowns on PGC migration has not been explored *in vivo*, frog and mouse PGCs readily migrate on mesentery explants (Heasman and Wylie, 1978) and mesoderm cells (Stott and Wylie, 1986), respectively, *in vitro*. A straightforward experiment to assess this dependency would be to manipulate cadherin function in these mesoderm cells and quantify PGC migration on them. Surprisingly, N-cadherin is not required in fly embryos for gastrulation or for several other morphogenetic events during developmental PGC migration (Schäfer et al., 2014). These mutant embryos provide an opportunity to assess whether somatic N-cadherin is necessary for PGC migration *in vivo*.

Potential new roles for somatic cadherins in PGC migration have recently emerged in fish and chick. Transplantation experiments showed that decreased E-cadherin expression levels in neighboring tissues negatively influence fish PGC migration, causing a loss or redirection of migratory polarity (Grimaldi et al., 2020). By contrast, migratory chick PGCs preferentially migrate on the cell-sparse, presumably less adhesive, side of the asymmetric dorsal mesentery after exiting the vasculature (Hen et al., 2014). This preference, however, was not noted in a prior study (Davis et al., 2008), and an asymmetric PGC distribution in the mouse dorsal mesentery, which has a similar left-right asymmetry (Davis et al., 2008), has not been observed. Ultimately, such preferences may not depend on cadherins and may arise from differences in ECM deposition or tissue stiffness, as discussed below.

As opposed to the defined roles somatic cadherins play in early-stage passive PGC translocation, the relative importance of somatic cadherins for active PGC migration has remained less clear cut. This is due to the challenge of perturbing cadherin function in a tissue-specific manner and the presence of an alternative substrate for migration, the ECM. More acute, targeted cadherin perturbations, such as with optogenetics (Ollech et al., 2020), combined with live imaging will be needed to clarify the role of somatic cadherins.

### Adhesions to the ECM

Somatic cells secrete and remodel ECM to delineate tissues and provide mechanical support for organogenesis and other functions (Frantz et al., 2010). Various groups have shown that ECM is present at the right place and time for use by actively migrating PGCs (Fujimoto and Yoshinaga, 1986; Heasman et al., 1981; García-Castro et al., 1997; Urven et al., 1989; Huss et al., 2019), and isolated PGCs readily attach to and migrate on fibrous interstitial ECM components, such as fibronectin (Heasman et al., 1981; De Felici and Dolci, 1989; Alvarez-Buylla and Merchant-Larios, 1986; Dzementsei et al., 2013), as well as basement membrane ECM, including laminin (De Felici and Dolci, 1989; Jaglarz and Howard, 1995), which is also prominent interstitially (Huss et al., 2019; García-Castro et al., 1997). Other structural ECM constituents, such as glycosaminoglycans (GAGs) and proteoglycans, are also present (Soto-Suazo et al., 2002), but their roles as adhesive substrates for migrating PGCs remain poorly defined. PGCs are also known to express integrin receptors (Table 1). Previous work has shown that mouse PGCs weakly adhere to purified GAGs (De Felici and Dolci, 1989). However, this does not exclude other functions for GAGs and proteoglycans, such as serving as sinks for chemokines (Sarris et al., 2012) and growth factors for PGC guidance.

Whereas cadherins directly promote PGC transport through cell–cell adhesions, ECM appears to play a more permissive role, demarcating a route for PGCs in frogs, chick and mice. Recent work has shown that the ECM is not homogeneous along the PGC path, particularly in the dorsal mesentery. In chick dorsal mesentery, fibronectin and hyaluronic acid are more enriched on the cell-sparse, right side, whereas sulfated GAGs show contralateral enrichment on the cell-packed, left side (Kurpios et al., 2008; Hen et al., 2014). Mouse dorsal mesentery has similar asymmetries in cell density and hyaluronic acid, although there is no current evidence that these PGCs demonstrate a preference for either side (Davis et al., 2008; Sanketi et al., 2022). As discussed above, based on the analysis of fixed samples, chick PGCs preferentially populate the right side of the dorsal mesentery (Hen et al., 2014), suggesting that chick PGCs could have a bias for hyaluronic acid or fibronectin as substrates, the altered mechanical properties of the right side, or a combination of these factors. Future studies assessing PGC migration in a dorsal mesentery with uniform ‘left’ identity through misexpression of *Pitx2* (Kurpios et al., 2008) may help better understand these effects.

An additional layer of regulation lies in the temporal expression of ECM along PGC paths. Fibronectin prominently appears in the dorsal mesentery during the most active phases of PGC migration in chick, frog and mouse; it then diminishes from the dorsal mesentery after PGCs have reached the gonadal anlage (Fujimoto and Yoshinaga, 1986), similar to laminin (García-Castro et al., 1997). The disappearance of these ECM components from the dorsal mesentery may prevent PGCs from backtracking once they have reached their target tissue. The emergence of the endoderm basement membrane fulfills a similar function in the frog, serving as a barrier to prevent PGCs from moving back after their egress into the dorsal mesentery (Heasman et al., 1985). PGCs further autonomously regulate their adhesion to ECM over time. Although the conclusions have been variable, mouse and frog PGC adhesion to fibronectin (Dzementsei et al., 2013; García-Castro et al., 1997; De Felici and Dolci, 1989) and laminin (García-Castro et al., 1997; De Felici and Dolci, 1989) generally decreases with developmental age *in vitro*, suggesting that PGC–ECM adhesion decreases as PGCs progress along the migratory route. This has been proposed to facilitate movement, as cell migration is known to be optimal at intermediate levels of adhesion (Palecek et al., 1997).

Altogether, the presence of ECM components along PGC paths, the cognate integrin receptor expression on PGCs and the demonstrated migration prowess of PGCs on these substrates *in vitro* create a compelling case that ECM is required for PGC migration *in vivo*. However, much of the current data are correlative in nature, and functional perturbations of PGC–ECM interactions in mice, fish, quails and flies, either through integrin ablation, matrix metalloprotease inhibition or ECM mutants, show only modest or no impairment of migration (Carver et al., 2021; Ismat et al., 2013; Huss et al., 2019; Anderson et al., 1999; Devenport and Brown, 2004; Kardash et al., 2010; Jaglarz and Howard, 1995). Collectively, these results indicate that PGCs may use other ECM receptors for migration, such as syndecans or discoidin domain receptors (Table 1) or that the ECM may play a more permissive role in creating an ideal mechanical environment for migration (Truszkowski et al., 2023). Yet another possibility is that PGCs may utilize a friction-based migration strategy that does not require specific adhesion (see below).

### Chemical cues

To migrate to the gonad, PGCs must respond to various chemical signals from the surrounding environment. Such cues can be attractive, repellent, or promote overall motility and preservation of PGC fate. Loss of any such cue results in PGCs failing to reach the gonad. One of the most studied chemical cues guiding PGC migration is C-X-C motif chemokine 12 (CXCL12, also known as stromal cell-derived factor 1, or SDF1a). This classic chemoattractant is broadly expressed in mammalian embryogenesis and adult tissues. Consistent with this, CXCL12 and its receptor, CXCR4, facilitate migration of many cell types, including stem cells, neurons, endothelial cells, hematopoietic cells, macrophages, lymphocytes, natural killer cells and cancer cells (Domanska et al., 2013). In many of these cell types, CXCL12 also impacts cell survival and proliferation and thus has crucial roles in development and oncogenesis (Guo et al., 2005; Wu et al., 2009; Guo et al., 2016). The PGC migration stage that requires CXCL12 and CXCR4 differs among species. In mice and frogs, CXCL12 and CXCR4 are required for the directed migration in the dorsal mesentery, but not for gut exit (Ara et al., 2003; Molyneaux et al., 2003; Stebler et al., 2004; Takeuchi et al., 2010). In zebrafish and medaka, in which PGCs do not enter the gut, CXCL12 is required throughout their migration to the gonad (Doitsidou et al., 2002), although in medaka CXCL12/SDF1a,b are more important for the early stages of PGC migration than for late-stage gonad colonization (Herpin et al., 2008). Chicken PGCs rely on CXCL12 to exit the vasculature and migrate to the gonad (Stebler et al., 2004). Together, these findings suggest that although the CXCL12/SDF1-to-CXCR4 axis is commonly required, the tissue and migration stage of this requirement varies among vertebrates (Table 2).

**Table 2.**
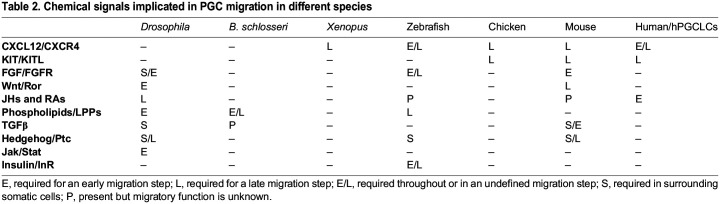
Chemical signals implicated in PGC migration in different species

Although CXCL12/SDF1 has been well-studied in PGC migration, it is not the only chemical cue that PGCs use. In fact, even though *CXCR4* mRNA is abundant in human PGCLC *in vitro* models (Mitsunaga et al., 2017), CXCR4 protein may not be present in migrating human PGCs *in vivo* (Gomes Fernandes et al., 2018). Another chemical cue commonly required in mammalian PGC migration and survival is kit ligand (KITL, also known as stem cell factor, or SCF, steel factor, or SLF, and mast cell growth factor; KITLG in human) (Table 2). Like CXCL12/SDF1, KITL is required for the migration, proliferation and survival of many cell types, including melanocytes, hematopoietic stem cells and PGCs (Wehrle-Haller, 2003; Keshet et al., 1991). In mice, KITL activates the KIT receptor tyrosine kinase in PGCs to promote their motility, proliferation and survival upstream of the apoptosis factor BCL2-associated X (Bax) (Stallock et al., 2003; Mccoshen and Mccallion, 1975; Runyan et al., 2006). KITL exists in soluble and membrane-bound forms that are widely and dynamically expressed along the migratory route of both mouse and human PGCs (Todaro et al., 2019; Cardoso-Moreira et al., 2019). KITL is expressed in the mouse endoderm at embryonic day (E) 7.25-8.0, then in the midline, and finally in the aorta-gonad-mesonephros; consistent with this, KITL loss compromises PGC speed, distance traveled, and gut internalization at E7.5, exit from the hindgut at E9.0, and gonad colonization at E11.5 (Keshet et al., 1991; Hoyer et al., 2005; Gu et al., 2009). Long postulated to promote motility rather than directionality, KITL does not function as an on/off migratory signal, as KITL displays haploinsufficiency and mutations in the KIT receptor cause PGC migration defects and apoptosis by E11.5 (Chen et al., 2013). Given these features and questions over CXCR4 presence, it will be interesting to determine the role of KITLG and KIT in human PGC migration.

Although KITL and CXCL12 have been well-studied, their requirements for PGC migration may be limited to vertebrate species. Indeed, CXC receptors and KIT ligands are chordate innovations (Bajoghli, 2013; Martinez-Morales et al., 2007; Grassot et al., 2006), yet PGCs migrate to the developing somatic gonad throughout the animal kingdom. A comprehensive survey that includes more species may provide greater insight into the chemical cues that PGCs use. An alternative approach is to assess requirements for more deeply conserved signaling cascades (Table 2). For example, Delta/Notch, Jak/Stat and TGFβ signaling cascades have been implicated in PGC migration; however, the directness of these requirements is unclear (Leotlela et al., 2007; Brown et al., 2006; Li et al., 2003; Godin and Wylie, 1991; Senft et al., 2019). Indeed, TGFβ may impact PGC migration indirectly by regulating collagen deposition in mouse (Chuva de Sousa Lopes et al., 2005) and Jak/Stat is required for sex-specific gonad development in flies (Wawersik et al., 2005). Manipulation of Hedgehog signaling has a profound impact on PGC migration in *Drosophila*, zebrafish and mice (Deshpande et al., 2001; Mich et al., 2009; Kim et al., 2020; Lee et al., 2023). However, the key Hedgehog response factor, Smoothened, is not required for zebrafish or fly PGC migration, and Hedgehog is not required for fly PGC attraction in response to known inducing factors (Mich et al., 2009; Renault et al., 2009; Kenwrick et al., 2019). One explanation for these contradicting observations is that Hedgehog, like other signaling cascades, is required for specification and patterning of the tissues PGCs encounter and use along their journey (Lee et al., 1992; Ma et al., 2002; Chen et al., 2020; Lee et al., 2023). Fibroblast growth factor (FGF) signaling is required for PGC migration in mice, zebrafish and flies, although in flies FGF signaling remodels the endoderm to allow PGC passage (Takeuchi et al., 2005; Chang et al., 2020; Pares and Ricardo, 2016; Seifert and Lehmann, 2012). In zebrafish, PGC migration is disrupted by both loss and gain of insulin-like growth factors (Sang et al., 2008). In fact, insulin dynamics underlie the effect of hypoxia on PGC migration in zebrafish (Lo et al., 2011). Insulin, although deeply conserved, has thus far only been shown to impact PGC migration in zebrafish, although PGC number is regulated by insulin in both zebrafish and chicken (Schlueter et al., 2007; Ye et al., 2023). Finally, Wnt signaling has been implicated in both mouse and *Drosophila* PGC migration. In mice, Wnt5 and its receptor Ror2 are required for mouse PGC migration (Laird et al., 2011; Chawengsaksophak et al., 2012). In *Drosophila*, the role of Wnt in PGC migration has been tied to lipid signaling, which itself has long been implicated in PGC migration (McElwain et al., 2011; Renault and Lehmann, 2006). Questions clearly remain regarding how each of these signaling cascades support PGC migration.

Across species, PGCs are highly sensitive to lipid metabolism and isoprenoid signaling (Steinhauer et al., 2009). Isoprenoids are produced by the mevalonate pathway (Santos and Lehmann, 2004; Thorpe et al., 2004), and inhibition of the rate-limiting mevalonate pathway enzyme HMGCR compromises PGC migration in mice, zebrafish and flies (Table 2) (Thorpe et al., 2004; Ding et al., 2008; Van Doren et al., 1998). In flies, the requirement for *Hmgcr* in PGC migration is linked to juvenile hormone (JH) synthesis enzymes (Santos and Lehmann, 2004; Barton et al., 2024). Like HMGCR, JHs induce and are necessary for PGC migration (Barton et al., 2024). JHs are structurally and functionally similar to retinoic acids (RAs), which were recently shown to transition human PGCLCs from a pre-migratory to a migratory program (Irie et al., 2023). RA synthesis enzymes are present in the surrounding somatic tissues in humans (Li et al., 2017; Irie et al., 2023), and RA induces mouse PGC migration *in vitro* (Barton et al., 2024). Together, these data suggest JH and RA isoprenoids may share a function in PGC migration in vertebrate and invertebrate species.

Sphingosine-1-phosphate (S1P) lipids have also been implicated in PGC migration. In *Botryllus schlosseri*, S1P and arachidonic acid facilitate germ cell migration *in vitro* and *in vivo* (Kassmer et al., 2015, 2020). In these colonial ascidians and in flies, PGC migration in response is facilitated by ABC transporters (Ricardo and Lehmann, 2009; Kassmer et al., 2020), which presumably are involved in release of these pro-migratory molecules. Degradation of bioactive lipids also play a central role in PGC migration, as lipid phosphate phosphatases (LPPs) are required for proper gonad colonization in *B. schlosseri*, zebrafish and flies (Kassmer et al., 2015; Paksa et al., 2016; Starz-Gaiano et al., 2001). The fly LPPs, Wunen and Wunen-2, are required for PGC survival and lateral sorting of PGCs into two populations that colonize each gonad (Hanyu-Nakamura et al., 2004; Renault et al., 2010; Starz-Gaiano et al., 2001). Interestingly, PGCs in these species migrate away from zones that express LPPs (Table 2) (Sano et al., 2005; Paksa et al., 2016) and catalytically active sites in Wunens are predicted to face the extracellular space, together suggesting that LPPs deactivate a chemical attractant and/or survival factor (Burnett and Howard, 2003; Slaidina and Lehmann, 2017). The identity of this pro-migratory/pro-survival molecule is not yet known, as Wunens and mammalian LPPs differ in PGC repulsion activity when ectopically expressed in *Drosophila*, yet are able to dephosphorylate many of the same lipids *in vitro* (Burnett and Howard, 2003; Burnett et al., 2004). Interestingly, when attractive cues and repulsive LPPs are co-expressed in flies or zebrafish, PGCs will approach, but not adhere to or enter, the attractive zone (Paksa et al., 2016; Kenwrick et al., 2019), suggesting that attractive cues act at long range, whereas repulsive cues act at short range. Whether repulsive cues facilitate PGC migration in mammals is unknown. Moreover, how PGCs integrate multiple signals is an interesting area of future exploration.

## The PGC flock: cue responses and intrinsic migration modes

### General principles of cell motility and polarization

Most metazoan cells have the latent capacity to polarize and migrate when placed in culture. In this 2D environment, cells generally move by extending thin, sheet-like, actin-filled protrusions, termed lamellipodia, away from the cell body. Polymerizing branched F-actin networks push and bulge the plasma membrane to create the characteristic lamellipodial morphology. The lamellipodium terminates in the lamella, a distinct region just proximal to the cell body filled with actin bundles of diverse architectures decorated with non-muscle myosin II (referred to as myosin II hereafter) (Case and Waterman, 2015).

The polarized addition of actin monomers at the leading edge of a migrating cell coupled with myosin II contraction in the lamella causes a net retrograde flow of the actin network toward the cell body. This rearward actin flow provides the motive force, much like the tread of a tractor, and no cell movement is possible if this flow is uncoupled from the underlying substrate. A cell can only move productively when the so-called ‘molecular clutch’ is engaged; that is, when the actin flow is transmitted to the substratum by transmembrane receptors (Case and Waterman, 2015). The canonical molecular clutch is composed of integrins, which cluster and assemble into focal adhesions to transmit actin flow to the ECM (Case and Waterman, 2015). However, diverse molecular clutches exist, including cadherins, which enable traction force generation on other cells (Yamada and Sixt, 2019).

Additional migration strategies exist in 3D environments, where cells are confined and pressed against their migratory substrates (Yamada and Sixt, 2019). Thus, although moderate, substrate-specific adhesion is absolutely required for 2D migration, 3D migration is possible with weaker, non-specific adhesion or even no adhesion at all (Paluch et al., 2016; Bergert et al., 2015). We hereafter refer to canonical, lamellipodia-driven migration as ‘mesenchymal’ and low-adhesion, rapid 3D migration as ‘amoeboid’. Generally, cells engaging in amoeboid migration have high levels of actomyosin contractility and low environmental adhesion compared with mesenchymal cells (Yamada and Sixt, 2019). Given their demonstrated migratory prowess on many different terrains, it is not surprising that recent work has shown that PGCs use a subset of amoeboid migration modes, some of which are considered substrate independent (Lin et al., 2022). Below, we summarize how PGCs move in 3D, focusing on how actin polymerization and actomyosin contractility are deployed to power movement, how the resulting motive forces are coupled to the environment, and how upstream signaling networks communicate to these molecular machines to direct movement.

### Actin polymerization and actomyosin contractility in PGCs

Early *in situ*, ultrastructural studies of migrating PGCs across animals noted that PGCs extend blunt pseudopods, but not lamellipodia, already hinting that a PGC moves very differently from a fibroblast in culture (Jaglarz and Howard, 1995; Clark and Eddy, 1975; Heasman and Wylie, 1978). These findings were later confirmed by dissociating embryonic tissues, isolating PGCs based on their unique morphological features, and using live imaging to assess their behavior on various substrates. When migrating on a cell-feeder layer or various ECM substrates, PGCs maintained an intriguing, rounded morphology during motility, with variable cell elongation along the axis of migration (Kuwana and Fujimoto, 1984; Wylie and Roos, 1976; Alvarez-Buylla and Merchant-Larios, 1986; Jaglarz and Howard, 1995). To our knowledge, only mouse PGCs extend fibroblast-like lamellipodia when seeded onto fibroblasts (Donovan et al., 1986). However, small pseudopods rather than lamellipodia are present in live migrating mouse PGCs in embryos (McDole et al., 2018), suggesting that lamellipodia may arise from particular experimental conditions.

Two crucial questions emerged from these observations: (1) how do PGCs move without lamellipodia, and (2) how can PGCs move across these varied substrates? Advances in imaging technology, the advent of green fluorescent protein (GFP), and a molecular understanding of PGC development, which enabled PGC-specific reporter expression and perturbations, have made it possible to address these questions through live imaging of PGC migration *in vivo* (Knaut et al., 2002; Wolke et al., 2002; Molyneaux et al., 2001; Sano et al., 2005).

**Fig. 3.**
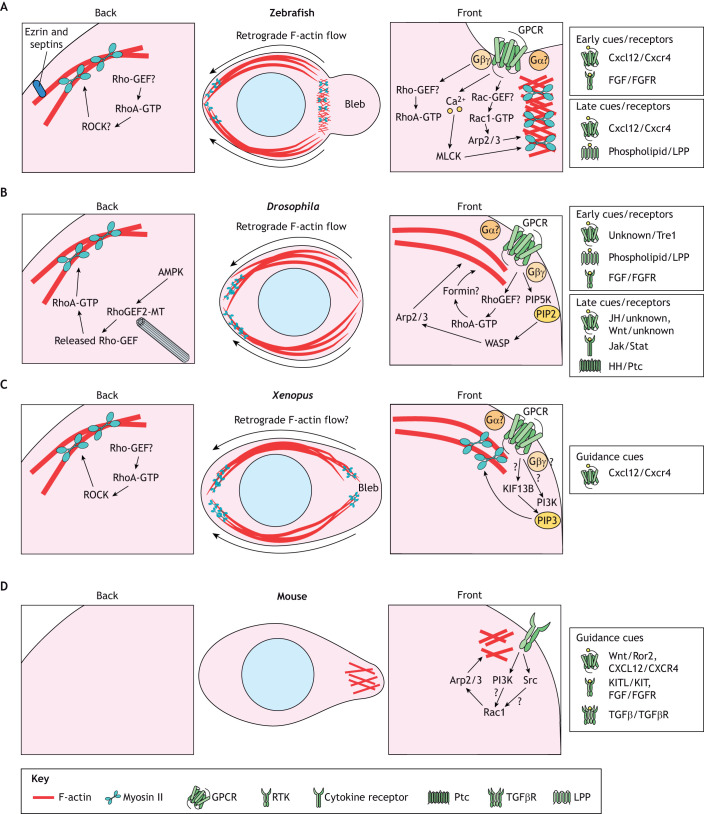
**PGC migration strategies and signaling logic.** Migrating PGCs interpret chemical guidance cues using different classes of receptors and lipid phosphate phosphatases. Rho-GTPases commonly establish cell polarity, but their upstream linkage to guidance receptors is largely unclear. Different receptor–cue pairs that affect PGC guidance (classified by a role in early or late migration if known, following the convention described in Table 1) are shown on the right of each species-specific PGC schematic. Lipid phosphate phosphatases (LPPs) dephosphorylate unidentified phospholipids to establish permissive migration zones. (A) Signaling pathways at the front and rear of zebrafish PGCs. In a typical front bleb cycle, Rac1 signaling first activates Arp2/3 to create an F-actin template. RhoA and calcium-MLCK signaling then drive actomyosin contractility to form a pressurized bleb, a region of plasma membrane detached from the actin cortex. Retrograde F-actin flow occurs from front to back, moving F-actin-linked proteins, such as Ezrin and septins, to the back to inhibit bleb formation and enhance front–back polarity. (B) Signaling pathways at the front and rear of *Drosophila* PGCs. Net actin polymerization at the cell front is required to maintain front-to-rear cortical actin flows, but the molecular details are unclear. A proposed front F-actin polymerization pathway includes the generation of PIP2 leading to Arp2/3-driven branched actin polymerization. However, inhibition of the Arp2/3 complex only mildly perturbs cortical flow, suggesting that a formin is involved. At the rear of PGCs, AMPK phosphorylates RhoGEF2, mediating its release from inhibitory microtubule interactions. This allows RhoGEF2 to activate RhoA and generate actomyosin contractility to direct cortical flow. (C) Signaling pathways at the front and rear of *Xenopus* PGCs. In culture, motile *Xenopus* PGCs are elongated with a leading bleb. Similar to *Drosophila* PGCs, RhoA signaling appears to be more crucial than Rac1 and is likely active at the rear. PIP3 has been shown to be enriched in blebs in a Kif13b-dependent manner and likely regulates actomyosin contractility to generate blebs. Retrograde F-actin flow likely occurs, but it is unclear whether it is local or global. (D) Signaling pathways at the front and rear of mouse PGCs. The importance of PI3K and Src are inferred from *in vitro* studies with inhibitors, whereas the importance of Rac1 has been shown *in vivo*. No functional data exists for signaling or cytoskeletal function at the rear of mouse PGCs. AMPK, AMP-activated protein kinase; Cxcl12, C-X-C motif chemokine ligand 12; Cxcr4, C-X-C motif chemokine receptor 4; FGF, fibroblast growth factor; FGFR, fibroblast growth factor receptor; GPCR, G protein-coupled receptor; HH, hedgehog; Jak, Janus kinases; JH, Juvenile hormone; KIF, kinesin family member 13B; LPP, lipid phosphate phosphatase; MT, microtubule; PI3K, phosphoinositide 3-kinase; PIP5K, phosphatidylinositol-4-phosphate 5-kinase; PIP2, phosphatidylinositol 4,5-bisphosphate [PtdIns(4,5)*P*_2_]; PIP3, phosphatidylinositol (3,4,5)-trisphosphate [PtdIns(3,4,5)P3]; Ptc, Patched; Ror2, receptor tyrosine kinase like orphan receptor 2; RTK, receptor tyrosine kinase; Src, Rous sarcoma oncogene; Stat, signal transducer and activator of transcription; TGFβ, transforming growth factor β; TGFβR, transforming growth factor β receptor.

High-resolution live imaging has revealed an elegant strategy that zebrafish PGCs use for efficient migration through various cellular terrains (Fig. 3A). Zebrafish PGCs alternate between phases of undirected and directed migration termed ‘tumbles’ and ‘runs’, whereby PGCs transiently lose and regain polarity, respectively (Reichman-Fried et al., 2004). In run phases, actin polymerization occurs at the cell front, and actomyosin contractility is enriched in two pools, one at the front and another at the cell rear (Olguin-Olguin et al., 2021; Blaser et al., 2006). A typical motility cycle occurs with the following sequence of events: (1) actin polymerizes at the cell front, serving as a template for actomyosin contractility, (2) front actomyosin contractility generates local hydrostatic pressure, causing cytoplasm to stream through ruptures in the actin cortex and inflate the plasma membrane, (3) bleb inflation, actin polymerization and rear actomyosin contractility cause a retrograde flow of cortical actin, moving various cortex-anchored proteins toward the rear, such as ezrin (Olguin-Olguin et al., 2021), and (4) the actin cortex reforms around the bleb and it retracts (Fig. 3A). Zebrafish PGCs can also extend mesenchymal-like, actin-filled pseudopods, but predominantly generate blebs unless surrounded by cells with high cortical tension (Truszkowski et al., 2023). Bleb generation likely reflects the PGC's internal state of actomyosin contractility, as isolated zebrafish PGCs continuously generate blebs when ­placed on glass coated with different ECM compositions or in a 3D Matrigel environment (Blaser et al., 2006). Extracted *Xenopus* PGCs (Terayama et al., 2013; Tarbashevich et al., 2011), chick PGCs under an agarose gel (Morita et al., 2023 preprint) and fly PGCs (∼20%) can spontaneously generate blebs (Lin et al., 2022), suggesting that PGCs innately exhibit high levels of actomyosin contractility. Although there is no substantial evidence that *Xenopus* or fly PGCs extend blebs *in vivo*, chick PGCs have been shown to extend a leading bleb when extravasating from vasculature (Morita et al., 2023 preprint).

In contrast to extensive deformation by extending pseudopods or blebs, fly PGCs maintain a spherical shape when moving *in vivo* (Fig. 3B). Like zebrafish PGCs, actin polymerization occurs at the front of directionally migrating fly PGCs, but actomyosin contractility is exclusively enriched at the cell rear (Lin et al., 2022). Rear actomyosin contractility exerts a local pulling force on the crosslinked cortical actin network, causing cell-scale retrograde cortical flow (Fig. 3B). Isolated *Xenopus* PGCs can also migrate with a consistent rounded shape, but are more elongated than fly PGCs and have a characteristic leading bleb (Terayama et al., 2013) (Fig. 3C), similar to migrating chick PGCs in Matrigel (Morita et al., 2023 preprint). It remains unclear whether mouse PGCs utilize a similar rounded migration mode (Fig. 3D).

Fly PGC movement becomes undirected at two stereotypical time points: (1) after endoderm egress before moving toward the mesoderm and (2) after reaching the general vicinity of the somatic gonadal precursors (Lin et al., 2022). During phases of undirected migration, cortical flows travel around the cell periphery, causing PGCs to spin in place (Lin et al., 2022). Keeping the motility system active when directionality is lost may enable a rapid response when appropriate guidance cues arrive. When fly PGCs are isolated and placed onto glass, around 40% of PGCs exhibit constitutive cortical flows (Lin et al., 2022). Fly PGCs, to our knowledge, are the only rounded amoeboid cells that display continuous cortical flows without external mechanochemical or genetic manipulation.

### Traction forces in PGC migration

**Fig. 4.**
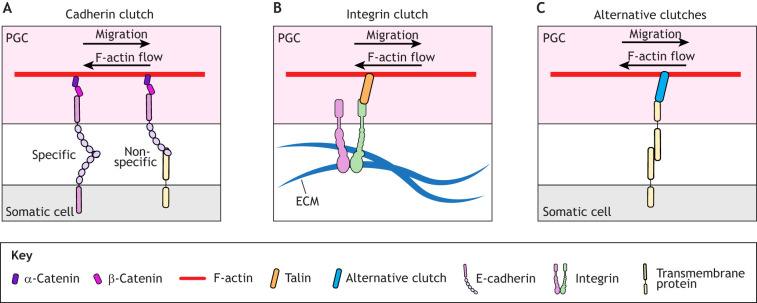
**PGC traction forces.** PGCs generate traction forces to migrate on other cells and ECM by coupling rearward F-actin flow to various molecular clutches, which transmit this force to the extracellular environment. Molecular clutches are adaptors that bind F-actin and are linked to transmembrane proteins either directly or indirectly. (A) Cadherin clutches. α-Catenin couples F-actin to cadherins and allows PGCs to migrate on other cells expressing the cognate cadherin or possibly another cadherin family member or a transmembrane protein that can transiently interact with the PGC cadherin. (B) Integrin clutches. Integrin clutches are well-described and allow cells to migrate on ECM. Talin serves as the molecular clutch between F-actin and ECM-binding integrin receptors. (C) Alternative clutches. Presumably, any protein that can interact with F-actin and a transmembrane protein can serve as a molecular clutch. These transmembrane proteins might generate traction by interacting with other transmembrane proteins or possibly allow migration via multiple non-specific environmental interactions. ECM, extracellular matrix.

Despite noted differences in the use of protrusions, fish, fly, and possibly frog, PGCs generate traction through a mechanism common to most migrating cells: the coupling of actin flow to a molecular clutch (Fig. 4). In contrast to the integrin-based molecular clutch used by mesenchymal cells (Fig. 4B), the fish and fly PGC clutch is likely cadherin-based (Jenkins et al., 2003; Kunwar et al., 2008; Kardash et al., 2010; Grimaldi et al., 2020) (Fig. 4A) and is well suited for cell-on-cell migration. Fly and fish PGCs show potent migration phenotypes when E-cadherin expression or function is compromised (Jenkins et al., 2003; Kardash et al., 2010; Kunwar et al., 2008), but not when integrin function is ablated (Devenport and Brown, 2004; Kardash et al., 2010). By contrast, integrin perturbations moderately affect mouse and avian PGCs (Anderson et al., 1999; Huss et al., 2019). This discrepancy may reflect the well-defined presence of ECM along the path of PGCs in mice and birds. Future experiments must address whether the fly PGC migration defects stem from disruption of a putative cadherin clutch or perturbation of directional sensing, as seen in collective cell-on-cell migration (Cai et al., 2014). Increasing or decreasing E-cadherin expression notably leads to defects in polarity maintenance in fish PGCs (Grimaldi et al., 2020). More generally, controlling appropriate cadherin expression levels is pervasive throughout PGC biology. In mice, frogs and fish, a decrease in E-cadherin adhesion accompanies active, solitary PGC migration (Blaser et al., 2005; Baronsky et al., 2016; Bendel-Stenzel et al., 2000). In contrast, although E-cadherin becomes enriched at the posterior of fly PGCs in clusters (Kunwar et al., 2008; LeBlanc and Lehmann, 2017), PGCs disperse without altering E-cadherin adhesion levels and can overcome enhanced cell–cell adhesion through ectopic E-cadherin expression (Lin et al., 2020).

Migratory molecular clutches are coupled intracellularly to F-actin flow and extracellularly to the environment (Fig. 4). The most likely intracellular component of the PGC cadherin clutch is α-catenin (Fig. 4A), which binds F-actin and forms a core complex with β-catenin and classical cadherins, such as E-cadherin (Halbleib and Nelson, 2006). In fly PGCs, the coupling between α-catenin and retrograde cortical actin flow is sufficiently strong to advect the core complex and lead to its accumulation at the rear of the cell (Kunwar et al., 2008), whereas, in migrating fish PGCs, E-cadherin remains uniformly distributed on the plasma membrane and requires ectopic increases in actin flow for rear transport (Blaser et al., 2006). Other cortex-anchored molecules that do not have extracellular components, such as Ezrin, Esyt2a and Septin9a, readily accumulate at the rear of fish PGCs in a flow-dependent manner to establish front-back polarity (Olguin-Olguin et al., 2021) (Fig. 3A). Actin flow-mediated transport of polarity effectors generally enhances migratory polarity (Maiuri et al., 2015), and it seems likely that many other proteins are moved in this manner.

Other molecular clutches may contribute to PGC migration; PGCs express many canonical cell–cell and cell–ECM adhesion molecules associated with migrating cells (Table 1) with known links to F-actin. Moreover, as described earlier, the cognate binding partners of these proteins, cadherins and ECM, are expressed along their migratory path. Therefore, PGCs are wholly capable of adhering to and generating traction on the diverse cells and ECM they encounter on their odyssey. Isolated frog PGCs can move in an adhesion-independent manner (Dzementsei et al., 2013). Thus, PGCs could couple actin flow to various molecules with extracellular domains to create non-specific friction for forward movement (Paluch et al., 2016) (Fig. 4C).

### Intracellular signaling in directional migration

Directed cell migration occurs when instructive signals position and maintain polarity effectors and cytoskeletal machinery along a front–back axis to drive persistent movement. Migrating cells sense and orient themselves towards diverse cues, from small molecules and lipids to electric fields. Of these wide-ranging cues, chemical cues are the best-described guidance signals for PGCs (Table 2). Transmembrane receptors, including receptor tyrosine kinases (RTKs) and G protein-coupled receptors (GPCRs), detect chemical cues and funnel signaling through a conserved set of effectors, including heterotrimeric G proteins, Ras and Rho-GTPases and phosphoinositides, to influence the cytoskeletal machines that power movement (SenGupta et al., 2021). Below, we describe the molecular pathways important for directed PGC migration, beginning with those that directly drive actin polymerization and actomyosin contractility.

The Arp2/3 complex and formins nucleate branched and unbranched actin filaments, respectively, and have well-described roles in cell migration. Branched F-actin networks efficiently push out a wide swath of the plasma membrane in lamellipodia, and linear F-actin filaments extend the membrane in finger-like protrusions called filopodia (Insall and Machesky, 2009). The Arp2/3 complex is activated by nucleation-promoting factors (NPFs), including WASP and Scar/WAVE, which themselves sit downstream of the small Rho-GTPase Rac1 (Insall and Machesky, 2009). Formins are activated by RhoA, another small Rho-GTPase (Insall and Machesky, 2009). RhoA also activates ROCK, a kinase that phosphorylates the regulatory light chain of myosin II to trigger actomyosin contractility (SenGupta et al., 2021). Directionally migrating cells typically create discrete zones of Rho-GTPase activity; in mesenchymal cells, active Rac1 at the front drives actin polymerization, whereas active RhoA at the rear promotes actomyosin contractility (SenGupta et al., 2021). The creation and maintenance of these Rac1 and RhoA zones relies upon a diverse family of RhoGEFs and RhoGAPs, which turn these small RhoGTPases ‘on’ and ‘off’, respectively (SenGupta et al., 2021).

Directionally migrating PGCs also exhibit polarized Rho-GTPase activity, but species-specific differences exist. Fish PGCs have elevated Rac1 and RhoA activity at the cell front and a separate region of RhoA activity at the rear (Kardash et al., 2010) (Fig. 3A). Another small Rho-GTPase, Cdc42, is also involved, but its activity profile remains unknown (Goudarzi et al., 2017; Jin et al., 2020). The RhoGEFs and RhoGAPs responsible for establishing these Rho-GTPase domains have not been uncovered, but creating localized Rho-GTPase activity is key, as globally expressing constitutively active (CA) or dominant-negative (DN) Rac1 or RhoA causes severe migration defects (Kardash et al., 2010) and optogenetic focal Rac1 activation is sufficient to specify the front of the PGC and guide cell movement (Olguin-Olguin et al., 2021). Mouse PGCs also rely on Rac1 signaling, and overall elevated Rac1 activity increases migration speed at the expense of accurate navigation (Jarysta et al., 2022) (Fig. 3D). Arp2/3 complex inhibition has been shown to promote bleb-based migration in cancer cells (Bergert et al., 2012), but its activation appears to be required for bleb-driven fish PGC migration, reflecting cell-type or environment-specific differences.

Fly PGCs, in contrast, have elevated RhoA activity at the rear but no apparent polarized Rac1 activity (Lin et al., 2020) (Fig. 3B). As expected, RhoA is vital for fly PGC migration; expression of CA or DN RhoA causes severe migration phenotypes (Kunwar et al., 2003), and optogenetic RhoA activation is sufficient to specify the cell rear and direct movement (Lin et al., 2020). CA RhoA causes a homogeneous increase in cortical actin density, whereas DN RhoA causes a thinning of cortical actin (Lin et al., 2022). Thus, the cortical flows that power fly PGC movement depend on the correct actin architecture. In contrast to fish PGC migration, branched actin polymerization appears to be dispensable for fly PGC migration, as the expression of DN Rac1 does not affect migration (Renault et al., 2010); DN Rac1 also has limited effects on frog PGC migration (Terayama et al., 2013) (Fig. 3C). Indeed, applying small molecules to inhibit the Arp2/3 complex only mildly perturbs cortical actin flow in fly PGCs, whereas formin inhibition depolymerizes the cortex, halting actin flow (Lin et al., 2022). The particular formins involved in fly PGC migration and how formin activity is stimulated at the front remain open questions.

Unlike our nuanced understanding of how small Rho-GTPases direct PGC migration, their upstream regulation and other putative pathways involved in navigation are less understood. Chemotactic RTKs and GPCRs include CXCR4 in fish, chick and frogs, KIT in mice, and the GPCR Tre1 in flies (Kunwar et al., 2003, 2008; Lin et al., 2020; Kim et al., 2021). These receptors signal through canonical pathways known to be important for navigation; for example, depletion of heterotrimeric G proteins causes similar defects to GPCR loss (Kunwar et al., 2008; Xu et al., 2012). Importantly, removing guidance does not affect basal, random cell motility, and PGCs can self-organize and move without any external cue (Olguin-Olguin et al., 2021; Lin et al., 2020, 2022). Future work will be needed to determine the molecular logic that connects these direct adaptors to Rho-GTPases.

Although our understanding of PGC guidance is limited, some additional players have been identified, and others may be ruled out. Phosphatidylinositol (3,4,5)-trisphosphate [PtdIns(3,4,5)*P*_3_; hereafter PIP3), an important regulator of the front of many chemotactic cells, is unpolarized in fly and fish PGCs (Kim et al., 2021; Xu et al., 2012), but is apparently enriched in frog PGC blebs (Tarbashevich et al., 2011) (Fig. 3C). Ca^2+^ levels are higher in the leading blebs of fish PGCs and drive actomyosin contractility through calcium-dependent kinases, such as MLCK (Blaser et al., 2006) (Fig. 3A). Additional molecular players include Rab10 (Mo et al., 2021), and others await further validation to determine their autonomy in PGCs.

## When migration fails: leveraging diverse fates of wayward PGCs among species

PGC migration is an essential step in reproductive development. However, the process is robust and to our knowledge no example has yet been identified in which PGC migration alone is compromised to the point where no PGCs colonize the gonad. In flies, a compensatory increase in PGC proliferation restores germline pool size (Gilboa and Lehmann, 2006), which may underlie a paucity of evidence that errors in PGC migration impact fertility. Moreover, fidelity of PGC migration to the gonad, and the fate of wayward PGCs, varies across species. In humans and mice, many PGCs fail to colonize the gonad in a normally developing embryo (Nguyen et al., 2019; Laird et al., 2011; Cantu and Laird, 2017; Stallock et al., 2003). PGCs found along the midline die by apoptosis (Runyan et al., 2006). In zebrafish *dead end* (*dnd1*) mutants, wayward PGCs adopt the fate of surrounding tissues (Gross-Thebing et al., 2017). In humans, mice and birds, wayward PGCs can form germ cell tumors (summarized by Nicholls and Page, 2021). Clinically, germ cell tumors vary based on developmental origin and potency states (reviewed by Oosterhuis and Looijenga, 2019). The ability of migratory PGCs to form multi-lineage teratomas has led to the hypothesis that germline commitment occurs not at PGC specification, but after PGCs have migrated and colonized the gonad (Nicholls et al., 2019). Whether PGCs that fail to properly migrate to the gonad are the same as PGCs that succeed needs further exploration. PGCs lacking the crucial germline factor Nanos fail to migrate to the gonad and adopt somatic fates in fly and frog embryos (Lai et al., 2012; Hayashi et al., 2004) and loss of the germline commitment factor Dead End (Dnd1) compromises PGC migration in zebrafish (Weidinger et al., 2003). These and other examples suggest that the maintenance of germline fate is required for proper migration. The diversity in fates of wayward PGCs across species may reflect differences in germline specification, developmental timing, and reliance on transcription or translation to regulate the germline proteome. Epidemiological studies and advances from diverse model organisms will be needed to acquire greater insight into genetic and non-genetic factors that affect the fate of wayward PGCs.

The different fates of wayward PGCs across model organisms can be leveraged to address different questions. For instance, the fact that all wayward, extragonadal PGCs in flies retain their identity and markers into late embryonic stages allows one to easily screen for genetic and environmental factors that compromise PGC migration (Barton et al., 2024; Moore et al., 1998). Female naked mole rats retain PGCs after birth (Brieno-Enriquez et al., 2023) and will be a great model to understand how PGC fate persists within the gonad and whether there are intrinsic differences in PGC migration, colonization timing and mutational load that lead to distinct downstream fates, as has been recently posited (Nguyen et al., 2019). PGC migration in classic and emerging developmental and regenerative biology model organisms, such as sea urchins, planaria and *Ciona*, are yielding insights into germline–somatic cell interactions (Campanale et al., 2014; Martik and McClay, 2015; Khan and Newmark, 2022; Takamura et al., 2002; Heikes et al., 2023). Investigations of PGC migration and development in agricultural pests and disease vectors are being leveraged to develop novel control methods (Ewen-Campen et al., 2013; Zhang et al., 2023).

Moving forward, animals with temperature-sensitive reproductive development, such as the red-eared slider turtle, may provide an opportunity to screen the non-genetic and environmentally sensitive factors that impact early PGC migration and development (Bachvarova et al., 2009; Czerwinski et al., 2016). Such investigations will not only increase our understanding of PGC migration across species and evolution, but may also lead to breakthroughs in ecology and disease vector fields.

## Conclusions, current challenges and the next frontier

Historically, the PGC migration field has been small. Yet the handful of labs focused on this key aspect of reproduction have made countless breakthroughs in our understanding of how PGCs respond to select informational cues to reach the gonad. The insights gained have changed the way we think about the migration of other cell types and have poised PGCs as an excellent model to understand cell migration *in vivo*. Indeed, findings of migratory cues and responses discussed in this Review can spur advances in our understanding of directed cell migration in various tissue terrains. We expect future insights will be fostered by the continued development of *in vivo* biosensors, microfluidic devices and live-imaging techniques. However, there are also challenges to overcome. Currently, there is a lack of genetic tools to specifically manipulate PGCs in most model organisms. This challenge persists because (1) PGCs are specified early in embryogenesis at a time when maternal RNAs and proteins are still present in many species, such as flies, frogs and fish, and (2) canonical PGC determinants and gonad-enriched factors are also expressed in pluripotent stem cells as well in the brain in animals ranging from flies to humans (Julaton and Reijo Pera, 2011; Kulkarni et al., 2020). One solution to this biological limitation will come from the analysis of PGCs and PGCLCs within *in vitro* assemblies and gastruloid models.

The generation of PGCLCs and subsequent *in vitro*-derived egg and sperm have rightly taken center stage in reproductive biology. Since Hayashi and Saitou's breakthrough generation of mouse PGCLCs and gametes (Hayashi et al., 2011, 2012), protocols have been developed to improve PGCLC survival and the efficiency of derivation (Overeem et al., 2023; Jo et al., 2022; Gell et al., 2020). PGCLCs are key to achieving translational goals, such as the preservation of endangered species and reproductive medicine (Hayashi et al., 2022; Wesevich et al., 2023). Other studies have more fundamental goals, such as to increase the number of model organisms used for scientific discovery (Jung et al., 2019; Seita et al., 2023) or to understand the underlying logic of human germline development (Saitou and Hayashi, 2021; Saitou, 2021; Irie et al., 2015; Gafni et al., 2013; Overeem et al., 2023). As these technologies develop, the need to validate the PGC-like nature of these cells is increasingly clear. One validation test recently proposed is PGC migration, which has the potential of being nondestructive (National Academies of Sciences and Medicine, 2023). However, the transition from pre-migratory to migratory profiles is a common bottleneck for human and non-human primate PGCLC models (Mitsunaga et al., 2017; Sasaki et al., 2015, 2016; Seita et al., 2023), which may be due to the use of ROCK1/2 inhibitors in specification protocols (Mitsunaga et al., 2017). Recent efforts aim to define factors that promote the migratory phase of PGCLC development, including CXCL12, activin and retinoids (Irie et al., 2023; Mitsunaga et al., 2017). Developmental progression of PGCLCs into the migratory phase can be aided by co-culturing with somatic gonadal cells or hindgut (Hwang et al., 2020; Yamashiro et al., 2018; Alves-Lopes et al., 2023; Oikawa et al., 2022). Alternatively, recent advances in the derivation of embryo models that develop through gastrulation provide a rich platform for modeling PGC migration (Cooke et al., 2023; Amadei et al., 2022; Tarazi et al., 2022). Regardless of approach, these new *in vitro* technologies are sure to provide ample hypotheses to test *in vivo*. These are surely exciting times to be studying the essential step in reproductive development: PGC migration.
